# Investigation of the Biocidal Performance of Multi-Functional Resin/Copper Nanocomposites with Superior Mechanical Response in SLA 3D Printing

**DOI:** 10.3390/biomimetics7010008

**Published:** 2022-01-02

**Authors:** Nectarios Vidakis, Markos Petousis, Emmanuel Velidakis, Nikolaos Mountakis, Dimitris Tsikritzis, Aikaterini Gkagkanatsiou, Sotiria Kanellopoulou

**Affiliations:** 1 Mechanical Engineering Department, Hellenic Mediterranean University, Estavromenos, 71410 Heraklion, Greece; vidakis@hmu.gr (N.V.); m.velidakis@hmu.gr (E.V.); mh90@edu.hmu.gr (N.M.); tm6759@edu.hmu.gr (A.G.); tm6751@edu.hmu.gr (S.K.); 2 Department of Electrical & Computer Engineering, Hellenic Mediterranean University, 71410 Heraklion, Greece; dtsikritzis@hmu.gr

**Keywords:** stereolithography (SLA), 3D printing, antibacterial, additive manufacturing (AM), copper (Cu), resin, mechanical, nanocomposites

## Abstract

Metals, such as silver, gold, and copper are known for their biocidal properties, mimicking the host defense peptides (HDPs) of the immune system. Developing materials with such properties has great importance in medicine, especially when combined with 3D printing technology, which is an additional asset for various applications. In this work, copper nanoparticles were used as filler in stereolithography (SLA) ultraviolet (UV) cured commercial resin to induce such biocidal properties in the material. The nanocomposites developed featured enhanced mechanical responses when compared with the neat material. The prepared nanocomposites were employed to manufacture specimens with the SLA process, to be tested for their mechanical response according to international standards. The process followed was evaluated with Scanning Electron Microscopy (SEM), Atomic Force Microscopy (AFM), energy-dispersive X-ray spectroscopy (EDS), and thermogravimetric analysis (TGA). The antibacterial activity of the fabricated nanocomposites was evaluated using the agar-well diffusion method. Results showed enhanced mechanical performance of approximately 33.7% in the tensile tests for the nanocomposites filled with 1.0 wt.%. ratios, when compared to the neat matrix material, while this loading showed sufficient antibacterial performance when compared to lower filler loadings, providing an added value for the fabrication of effective nanocomposites in medical applications with the SLA process.

## 1. Introduction

Microbial contamination of air, water, and soil by a variety of microorganisms produces problems in both living and non-living things, as well as in public health and industry. Antibiotic resistance genes have thus become more frequent in a wide range of microorganisms, including people and animals [1]. The host defense peptide (HDP) has mechanisms to inhibit or kill bacteria, and their physicochemical characteristics are imitated by various materials, such as copper, silver, titanium, and zinc, which exhibit antibacterial efficacy [1,2]. Metal Nanoparticles (NPs) are the most promising in this field, having demonstrated considerable antibacterial characteristics and being more commonly used in industry [3]. Still, the mechanisms underlying metallic nanostructures’ biocidal action are not fully understood [4].

The development of additive manufacturing (AM) has gained interest from both academic and industrial perspectives [5,6]. In the past decades, a wide range of AM techniques have been invented and commercialized, utilizing a variety of polymeric, composite, and metal materials [7,8,9]. Among the most known and those with enough impact in industrial applications technologies are Fused Filament Fabrication (FFF), stereolithography (SLA), and Selective Laser Sintering (SLS) [10,11,12,13]. All these technologies differ in their technical parameters and utilized materials; however, they all share the same operating principle of layer-by-layer manufacturing [14,15]. While FFF has gained the greatest market share and interest in composite material development, SLA is gaining continuous advancement [16,17].

SLA 3D printing technology is an AM method that utilizes resins in liquid form, which are introduced into a tank with a transparent bottom [18,19,20]. The laser light is electronically driven through reflective mirrors to the bottom of the tank [21,22,23]. The laser spot follows a path specified by the software at each layer. This introduction of light enables the polymerization of the photosensitive resin, and the process is repeated to fabricate each layered component [17,21,24,25]. As a result of the SLA 3D printing method, the fabricated parts are solid, in contrast to the typically hollowed parts of other 3D printing methods [26,27,28]. The laser spot size enables the fabrication of micro-sized layers, usually ranging from 25 to 100 µm, resulting in the manufacturing of parts with smooth and consistent surface and structure [23]. Additionally, the specifications of the SLA 3D printing process enhance the capability of manufacturing complex geometry structures [12,29].

SLA, followed by AM techniques such as DLP and LCD are mostly utilized in medical, dental, and coherent implementations [21,22]. Prototyping applications of sectors, such as automotive and aerospace, also utilize SLA technology; however, in dental and medical utilization, components are additionally fully operational [30,31]. The recent pandemic has led to a wide range of problems in many economic sectors, including the sensitivity of the supply chain to such situations [26,32]. The worldwide 3D printing community has exhibited the forward momentum of AM by reducing the deficits, and in many cases, eliminating them [32,33]. Sufficient case studies have been presented regarding the manufacturing of face shields and respiratory components during this period [34,35], initiating a potential for further 3D printing implementations in the medical sector [11,36].

Medical, dental, and other similar applications require excellence in design, manufacturing, quality control, and the entire production process [37,38,39]. Most strict regulations worldwide are introduced for medical devices, and the materials used in such applications are evaluated in depth for their reliability, safety, and effectiveness [39]. The development of such composite materials to be introduced in SLA AM technology could enable the development of stiff reliable composites with advanced electrical conductivity properties [40,41,42], antibacterial performance [14,31,43], etc. In this way, the manufacturing capabilities would be greatly increased, while the manufacturing cost could be reduced with the introduction of AM technology.

Nanotechnology can introduce such specifications when used in composites fabrication [44]. Extensive research has been conducted on the introduction of nanoparticles in various forms, sizes, and combinations [40,45,46]. Nanocomposites have been widely introduced in FFF materials [13,14,47,48], while the development of nanocomposites in SLA materials is still not widely investigated [12,13,23]. Copper (Cu) is a metallic material that has been used in several applications over the years [49,50,51]. Its ductile behavior and antibacterial properties introduced a definite advantage for composites’ development. Copper nanoparticles have been introduced in matrix materials for nanocomposites’ fabrication, for catalysts due to large surface-to-volume ratio, antibacterial surfaces, and electrical applications [52]. Sufficient research has also been conducted on AM, mainly in materials for the FFF process [53,54,55,56].

This work is an attempt to develop materials suitable for SLA 3D printing, mimicking the properties of HDPs, to be used in engineering and medical applications. Copper’s biocidal properties were used to accomplish this. A common ultraviolet (UV)-cured photosensitive SLA resin was used as a matrix material, and it was filled with copper (Cu) nanoparticles at various loadings. Low filling ratios were selected for the study. The method used common laboratory equipment to investigate the potential of nanocomposites’ fabrication. To the best of the authors’ knowledge, no similar study has been presented in literature so far on the manufacturing of SLA nanocomposites with copper nanofillers for plausible implementation in medical applications. The antibacterial performance of all the nanocomposites developed was verified with the laboratory process followed in this work. Additionally, the nanocomposites developed exhibited enhanced mechanical performance when compared with the matrix material. Specimens were manufactured with the SLA process and assessed according to international standards. Their mechanical, thermal, and morphological properties were studied. Thus, the novelty of the work is related to the process followed, and the nanocomposites developed, with a common low-cost commercial UV resin for the SLA 3D printing process, which can be exploited in various demanding applications, requiring enhanced mechanical properties in combination with antibacterial performance. The results show the potential of the commercial SLA UV-cured resin Cu nanocomposites, as the mechanical performance was enhanced in all filler loadings studied.

## 2. Materials and Methods

**Figure 1:**
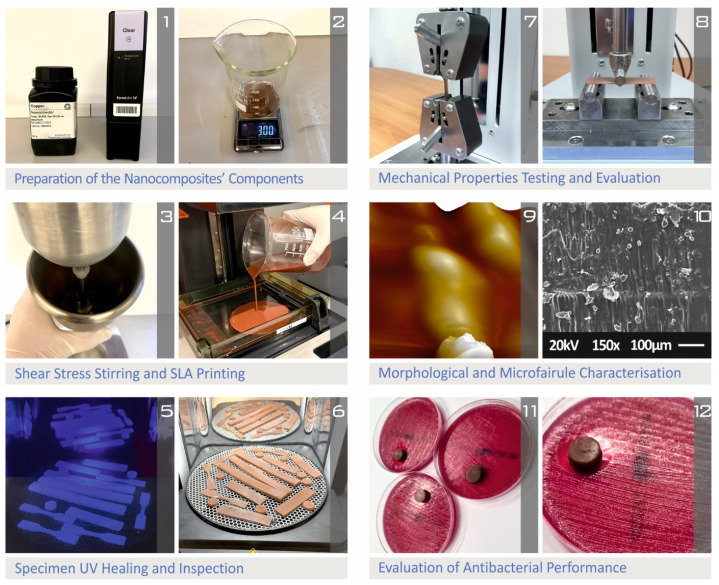
Workflow presentation of the followed processing using images captured during the study.

Figure 1 summarizes the steps followed in this work for the preparation of the nanocomposites, the manufacturing of the specimens, and their characterization processing.

### 2.1. Materials

The matrix material selected for the current study was Formlabs Standard Clear V4 (Formlabs Inc., Somerville, Massachusetts, United States) procured from a local supplier. Standard clear V4 resin (SC) is a commercially available product, and according to the safety data sheet consists of 55–75% urethane di-methacrylate, 15–25% methacrylate monomer(s), and less than 0.9% diphenyl (2,4,6-trimethybenzoyl) phosphine oxide. Copper nanoparticles (Cu) were used as fillers for the nanocomposite fabrication. Cu nanopowder was procured from Nanografi (Nanografi Inc., Ankara, Turkey), and the size of the nanoparticles ranged from 80 nm to 240 nm with a purity of 99.95%.

### 2.2. Nanocomposites and Specimens Fabrication

**Figure 2:**
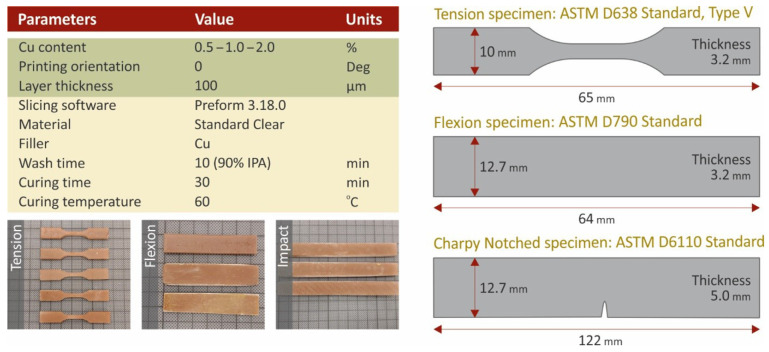
Fundamental SLA 3D printing settings, specimens’ dimensions, and images from 3D printed specimens.

Nanocomposites were prepared using a high-rotational-speed laboratory mixer, which enabled high shear forces during the mixing procedure. Cu nanoparticles were weighed and introduced into the SC resin matrix material at filling ratios of 0.5 wt.%, 1.0 wt.% and 2.0 wt.% respectively. The mixing process of each nanocomposite had a duration of 30 min to achieve the optimum dispersion of the nanoparticles in the nanocomposites. Before the uncured nanocomposites were poured into the 3D printer tank, they were degassed using a laboratory vacuum chamber. An SLA 3D printer was used for the specimens’ fabrication. Formlabs Form 2 (Formlabs Inc., Somerville, Massachusetts, United States), equipped with a resin tank of Formlabs Tank LT, was employed, which is a 3D printer equipped with a laser light source with a wavelength of 450 nm and a laser spot of 150 µm. In Preform software version 3.16 (Formlabs Inc., Somerville, Massachusetts, United States), the necessary G-code file and layer height setup were set at 100 µm. All specimens were placed and oriented so that the widest side had a direct touch on the build platform, while for the light source setting, the default setting for the matrix material was used for all studied nanocomposites. The 3D printing process was followed by thorough washing of the specimens in an isopropyl alcohol (IPA) bath with 90% purity, in a Formlabs Form Wash machine for 10 min. The specimens were washed and thoroughly dried in room conditions (22 °C, 50% RH). Then they were placed in a UV curing chamber, i.e., a Formlabs Form Cure (Formlabs Inc., Somerville, Massachusetts, United States) machine. Surface curing of the specimens was conducted for 30 min at 60 °C, according to the manufacturer’s specifications for the matrix material. Figure 2 shows the fundamental 3D printing settings used in this work.

### 2.3. Mechanical Performance Testing

Tensile, flexural, impact, and Vickers microhardness measurements were performed to evaluate the mechanical performance of the studied materials. The tensile properties were studied according to the ASTM D638-02a international standard on five (5) type V specimens of 3.2 mm thickness. For this purpose, an Imada MX2 (Imada Inc., Northbrook, IL, USA) machine equipped with standardized grips was used. The tension speed was set to 10 mm/min, while the tests were conducted in room conditions (21 °C, 50% RH). The Imada MX2 machine was also used for the flexural tests. The grips were replaced with the flexural setup of three-point bending (with 52 mm span), following the ASTM D790-10 international standard. For the flexural test, five specimens of 3.2 mm thickness were evaluated. The remaining specimens’ dimensions are shown in Figure 2. Charpy’s notched specimens impact tests were conducted according to ASTM D6110-04 international standard. Five (5) specimens were assessed using a Terco MT220 machine (Terco AB, Huddinge, Sweden). Randomly selected specimens were used for Vickers microhardness measurements according to the ASTM E384-17 international standard, which was followed. Five (5) measurements were taken on each case.

### 2.4. Morphological, Thermal, and Antibacterial Analysis

To investigate the processing efficiency, Scanning Electron Microscopy (SEM) analysis was conducted on the fractal and side surfaces of 3D printed tensile specimens. A JEOL 6362LV (Jeol Ltd., Norwood, MA, USA) apparatus was used for this purpose, while images were taken on two (2) magnification levels. Samples were randomly selected and first sputter-coated with gold (Au) to avoid charging effects. The electron microscope was set in the high vacuum mode at 20 kV acceleration voltage. The same apparatus was used for energy-dispersive X-ray spectroscopy (EDS) analysis on uncoated specimens to verify the main elements in each material.

Atomic Force Microscopy (AFM) was used to investigate specimens’ surface morphology and roughness in the micro-scale. A microscope solver P47H Pro (NT-MDT, Moscow, Russia) apparatus was used for this purpose. Commercially available silicon cantilevers with a scanning frequency of 1 Hz, cantilever spring constant of 35 N/m, tip cone angle of 20°, and tip radius of 10 nm were used at a resonant frequency of 300 kHz. Samples of approximately 10 mg from the specimens were also assessed for their thermal behavior by thermogravimetric analysis (TGA). A Perkin Elmer Diamond TGA/DTGA (Perkin Elmer Inc., Waltham MA, USA) apparatus was used, and measurements were taken at a temperature range from 40 °C to 550 °C. The temperature ramp was set at 10 °C/min.

The antibacterial activity of the fabricated nanocomposites was investigated using the agar well diffusion method. Tests were conducted in a microbiological laboratory for two (2) different bacteria. Gram-negative Escherichia coli (*E. coli*) and gram-positive Staphylococcus aureus (*S. aureus*) were cultivated in 85 mm diameter Petri dishes. The tested cylindrical specimens were 3D printed according to the specifications shown in Figure 2, and their dimensions were 12.7 mm in diameter and 5.00 mm in height. Petri dishes were placed in an oven at 37 °C for a period of 24 h targeting the optimized diffusion of the antimicrobial agents in the agar and inhibiting the germination and growth of the test microorganism. Subsequently, the inhibition zones developed peripherally of the 3D printed specimens were measured using optical equipment.

## 3. Results

### 3.1. Mechanical Performance Analysis

**Figure 3:**
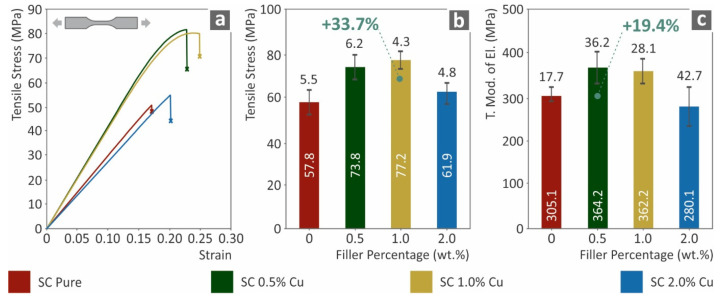
(**a**) Typical tensile stress (MPa) to strain (mm/mm) curves, (**b**) tensile stress at break (MPa) to filler loading (wt.%), (**c**) tensile elastic modulus (MPa) to filler ratio (wt.%).

The tensile test results are shown in Figure 3. Figure 3a presents a typical curve of tensile stress (MPa) versus strain (mm/mm) for each tested nanocomposite. The introduction of Cu nanoparticles in low rations has a clear effect on the ductility of the nanocomposites. The ductile behavior of copper affected the brittle behavior of the SC resin for all cases studied, except SC Cu 2.0 wt.%. Such a change can be attributed to saturation effects or low-quality polymerization processing, due to the increased filler loading. Figure 3b,c show the tensile stress at break (MPa) and tensile modulus of elasticity (MPa) to filler ratio (wt.%). Figures showed an enhancement effect from the introduction of Cu nanoparticles in the SC Cu 0.5 and 1.0 wt.% nanocomposites, while a similar degradation effect is shown for the SC Cu 2.0 wt.%, which is consistent with the stress to strain curves of Figure 3a.

**Figure 4:**
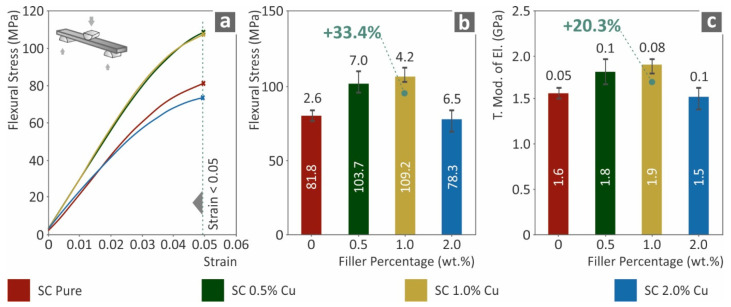
(**a**) Typical flexural stress (MPa) to strain (mm/mm) curve, (**b**) Flexural stress (MPa) at 5.0% strain (following the standard instructions) to filler loading (wt.%), (**c**) flexural elastic modulus (MPa) to filler ratio (wt.%).

The flexural test results are shown in Figure 4. As shown in Figure 4b,c, both the flexural strength (MPa) at 5.0% strain and the flexural modulus of elasticity were significantly increased compared to those of the pure SC material, which is consistent with the tensile test results. SC Cu 1.0 wt.% nanocomposite presented the highest flexural performance among the cases studied and especially a 33.4% increase was measured at the flexural stress and a 20.3% increase at the flexural modulus of elasticity. SC Cu 2.0 wt.% nanocomposite presented vigorous degraded flexural strength and modulus of elasticity, which also agrees with the tensile test results. Filling ratios higher than 1.0 wt.% enabled plausible agglomerations in the nanocomposite, which could plausibly deteriorate mechanical performance. Additionally, this degradation in the mechanical performance at higher loadings could also be attributed to low polymerization of the material, due to the high concentration of copper, which could enhance the diffusion effect during 3D printing.

**Figure 5:**
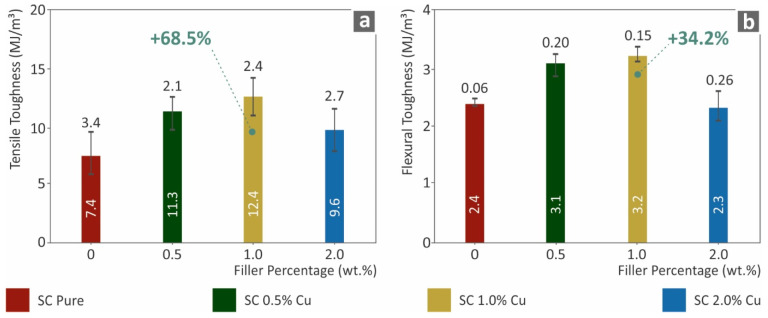
(**a**) Average tensile toughness (MJ/m^3^) to filler loading (wt.%), (**b**) Average flexural toughness (MJ/m^3^) to filler ratios (wt.%).

Figure 5 shows the results for the tensile toughness (MJ/m^3^) and the corresponding flexural toughness (MJ/m^3^). These measures were calculated as an integral of the corresponding stress–strain curves of all the tested specimens, and the average values are presented below. Such toughness calculations show a generic view of the absorbed energy during the tests. Following the tensile and flexural performances, the corresponding toughness of the nanocomposites assessed was enhanced in the case of SC Cu 1.0 wt.% nanocomposite. An increase of 68.5% was calculated for tensile toughness in comparison to the neat SC material, while the same material absorbed 34.2% more flexural energy in comparison to pure SC materials. Increasing the filler ratio over 1.0 wt.% showed a decrease either in tensile or in flexural properties. Such a decrease could plausibly occur owing to low polymerization effects. Plausible agglomerations could also introduce such behavior. These phenomena are more thoroughly analyzed in the discussion section of this work.

**Figure 6:**
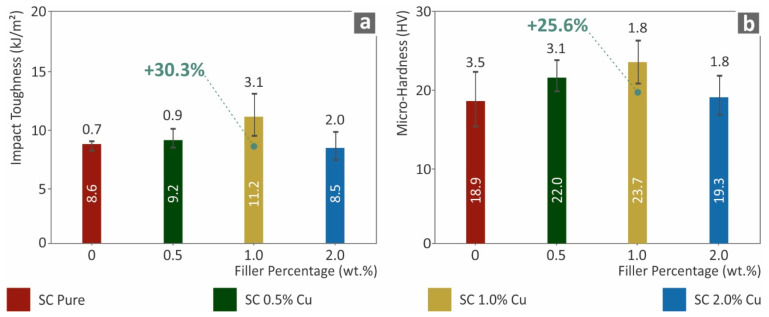
(**a**) Charpy’s notched impact toughness (kJ/m^2^) to filler loadings (wt.%), (**b**) Vickers microhardness (HV) to filler ratios (wt.%).

The mechanical performance analysis was also investigated with Charpy notched impact tests and Vickers microhardness measurements, the results of which are presented in Figure 6. In agreement with the other mechanical tests of this work, the impact toughness increased by approximately 30% for SC Cu 1.0 wt.% nanocomposite in comparison to pure SC resin. The ductile behavior of the Cu nanoparticles enabled the fabrication of nanocomposites that absorb higher energy levels, which increased the impact strength on the specimens. Similar performance was seen for the microhardness of the surfaces, which was approximately 25% higher than that of the neat material. In general, the mechanical performance results were in good agreement. Lower filler loadings (0.5 wt.% and 1.0 wt.%) showed an increasing effect, while the highest SC Cu 2.0 wt.% nanocomposite had the lowest performance in all mechanical tests conducted.

### 3.2. Thermal, Morphological, and Antibacterial Analysis

#### 3.2.1. Thermal Analysis

**Figure 7:**
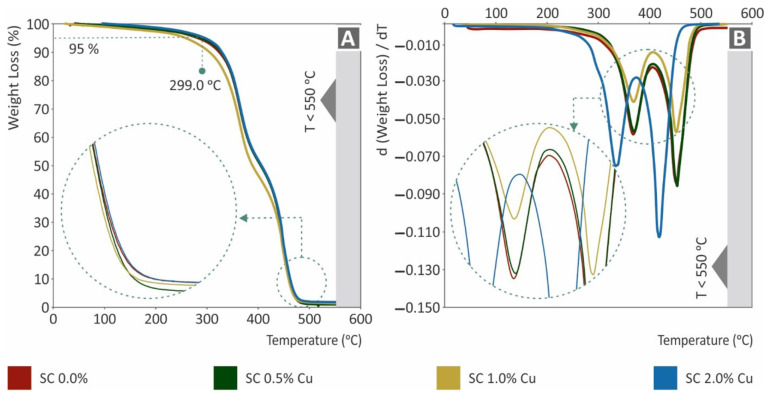
(**A**) Sample’s weight (%) to temperature (°C), (**B**) weight loss rate (mg/mg) to temperature (°C).

The TGA results are shown in Figure 7. Specifically, in Figure 7A, the weight (%) of each nanocomposite to temperature (°C) is presented, while in Figure 7B, the weight loss rate is presented in comparison to the corresponding temperature. The remaining nanocomposites were in good agreement with the corresponding filling ratios. Considering the effect of copper, which reduced the mass reduction rate, as expected, it could be assumed that the mixing procedure was of good quality. In agreement with the results of other tests, the SC Cu 2.0 wt.% nanocomposite showed vigorous degradation. As the effect of copper nanoparticles could not be responsible for such performance, a plausible implication of low-quality polymerization could be assumed through the TGA graphs.

#### 3.2.2. Morphological Analysis

**Figure 8:**
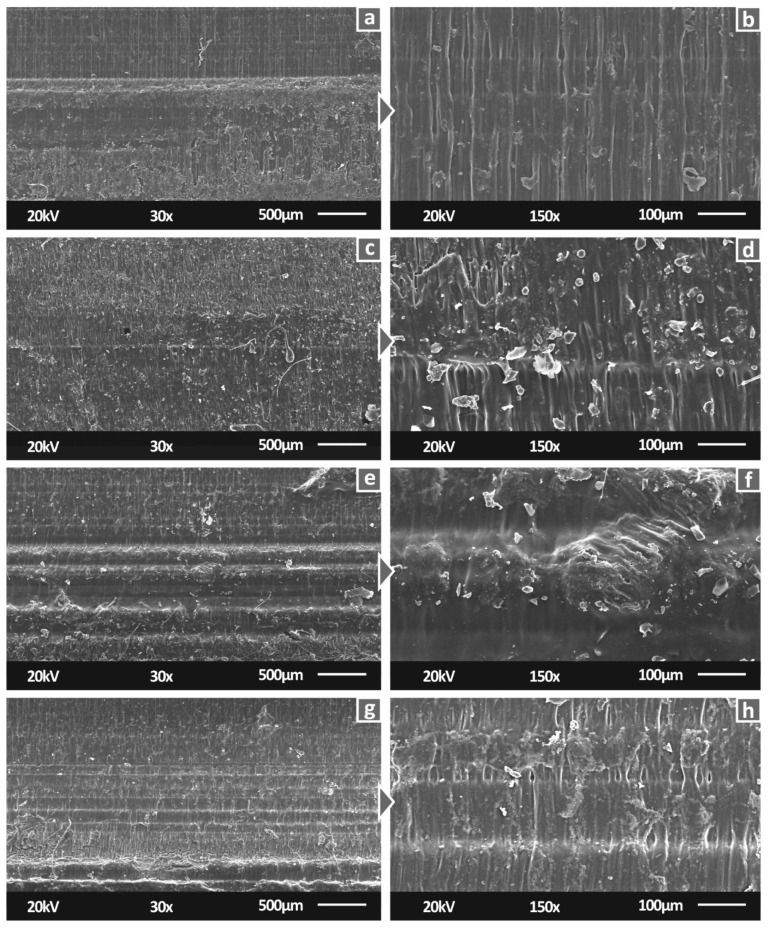
Side surface of tensile specimens in 30× magnification for (**a**) Pure SC, (**c**) SC Cu 0.5 wt.%, (**e**) SC Cu 1.0 wt.%, (**g**) SC Cu 2.0 wt.%, same surfaces in 150× magnification for (**b**) Pure SC, (**d**) SC Cu 0.5 wt.%, (**f**) SC Cu 1.0 wt.%, (**h**) SC Cu 2.0 wt.%.

Figure 8 shows the side surfaces of tensile specimens for all studied materials. 30× magnification level images verified the 3D printing specifications. Specifically, neat SC and SC Cu 0.5 wt.% materials have a smooth surface, indicating a fine interlayer fusion. A similar view is also shown for SC Cu 1.0 wt.% nanocomposite. Fusion is in fine agreement with minor differences which could plausibly imply slight agglomerations. In the case of SC Cu 2.0 wt.% nanocomposite, dimensional accuracy of layering shows a rather fine quality, while the slightly more intense visible layers could assume lower interlayer fusion. As per SLA technology, due to the operating principle, interlayer and intralayer fusion quality depend on the polymerization process quality. Thus, a low-quality polymerization assumption could plausibly be assumed for high filling loadings. Figure 8a,b, showing the side surface of tensile specimens built with pure resin show a smoother external surface of the specimens, typical for this type of manufacturing process, attributed to proper polymerization and correct 3D printing settings. With the addition of 0.5 wt.% filler (Figure 8c,d), a similar surface pattern appears, but with abnormalities, in this case, attributed to low polymerization of the material during the process. Increasing further the filler loading to 1 wt.% (Figure 8e,f) the pattern of the side surface changes. The abnormalities are reduced, and the build layers are more clearly visible. At the highest filler loading tested in this work (Figure 8f,h—2 wt.%), specimens’ side surface built-quality seems to be reduced, when compared to lower filler loadings.

**Figure 9:**
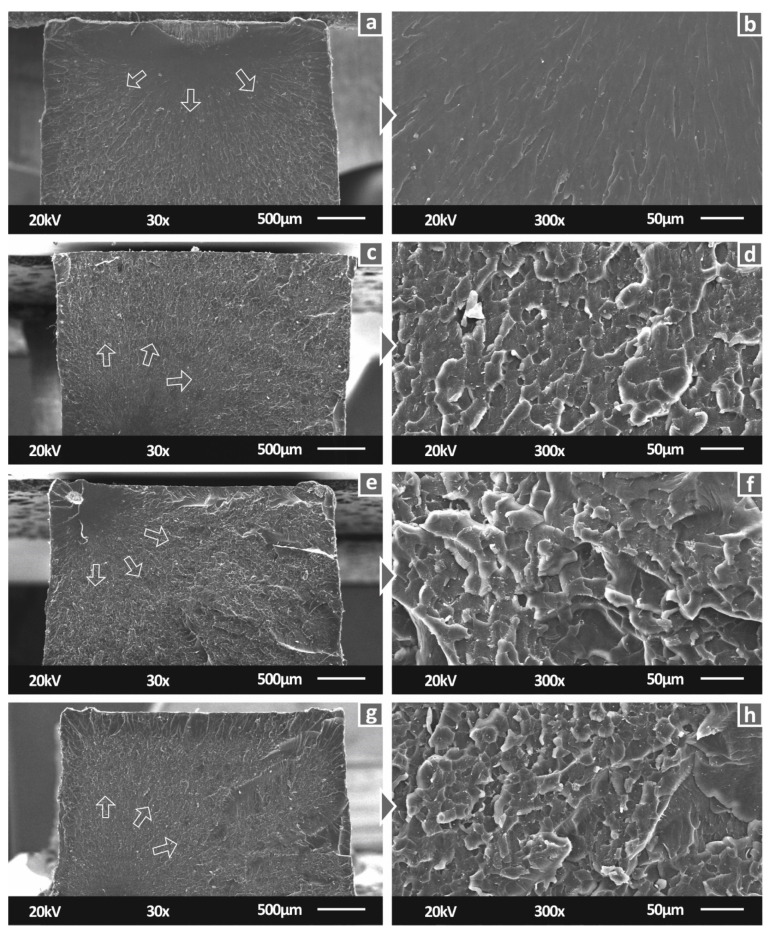
Fracture surface of tensile specimens in 30× magnification for (**a**) Pure SC, (**c**) SC Cu 0.5 wt.%, (**e**) SC Cu 1.0 wt.%, (**g**) SC Cu 2.0 wt.%, same surfaces in 300× magnification for (**b**) Pure SC, (**d**) SC Cu 0.5 wt.%, (**f**) SC Cu 1.0 wt.%, (**h**) SC Cu 2.0 wt.%. Arrows in the pictures show the fracture evolution in the section.

Figure 9 shows the fractal areas of tensile specimens for all studied materials. The lower magnification images of the tensile specimens’ fracture area provided sufficient information on the fracture mechanism. In particular, the introduction of Cu nanoparticles enabled a more ductile performance on the fabricated nanocomposites, which is observed at the fracture surfaces. The sudden brittle break of the neat SC specimen can be attributed to the sharp surface presented in the corresponding image (Figure 9A). Nanocomposites with filler loading of 0.5 and 1.0 wt.% showed a smaller region that failed in a brittle way, which can be observed at the start of the fracture arrows shown in the figure. The remaining specimen’s area shows a more ductile fracture mechanism, which is in good agreement with the corresponding mechanical analysis. In higher-magnification images, the ductile behavior is visible, while significant agglomerations cannot be seen.

**Figure 10:**
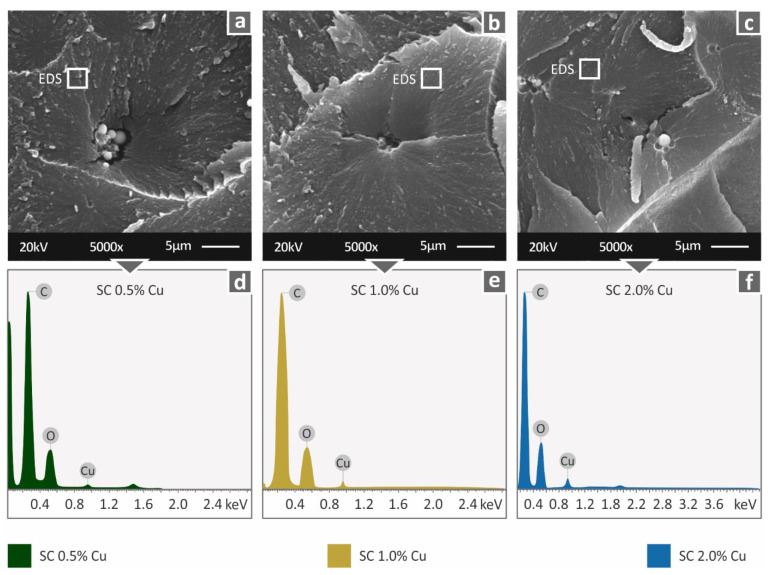
Fracture area high magnification captures at 5000× level (**a**) SC Cu 0.5 wt.%, (**b**) SC Cu 1.0 wt.%, (**c**) SC Cu 2.0 wt.% and the corresponding EDS analysis results (**d**) SC Cu 0.5 wt.%, (**e**) SC Cu 1.0 wt.%, (**f**) SC Cu 2.0 wt.%. White squares indicate the areas for EDS analysis.

Higher magnification images were taken in the fractal area (Figure 10a–c). At the highest magnification level, slight agglomerations were observed, which did not create any processing problems, although the implications of the reduced polymerization quality at higher loadings still exist. EDS analysis (Figure 10d–f) verified the presence of copper in the materials, and graph peaks reveal an indication of good dispersion of the filler in the matrix material.

**Figure 11:**
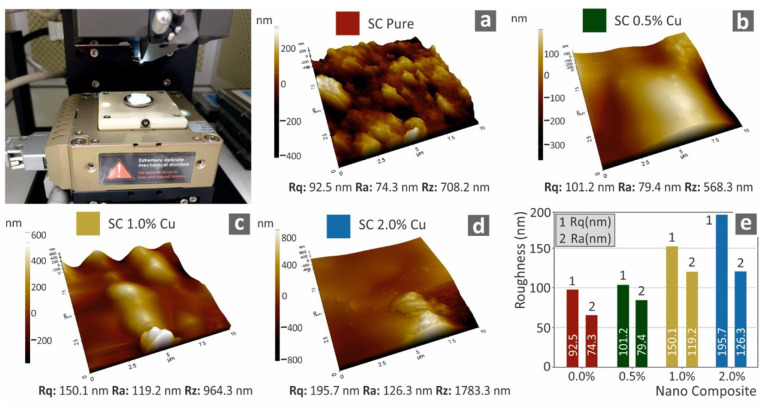
Three-dimensional graphical presentation of the measured with AFM surfaces of 3D printed specimens for all the tested materials (**a**–**d**), (**e**) measured roughness (nm) to filler ratio (wt.%).

Finally, through AFM analysis of the cured 3D printed specimens’ surfaces, their morphology is further investigated. Figure 11 presents three-dimensional images of the specimen surfaces captured with AFM, along with the corresponding average calculated roughness of each studied nanocomposite. The increase of the filler, as expected, further enhances the deterioration of the surface quality.

#### 3.2.3. Antibacterial Analysis

**Figure 12:**
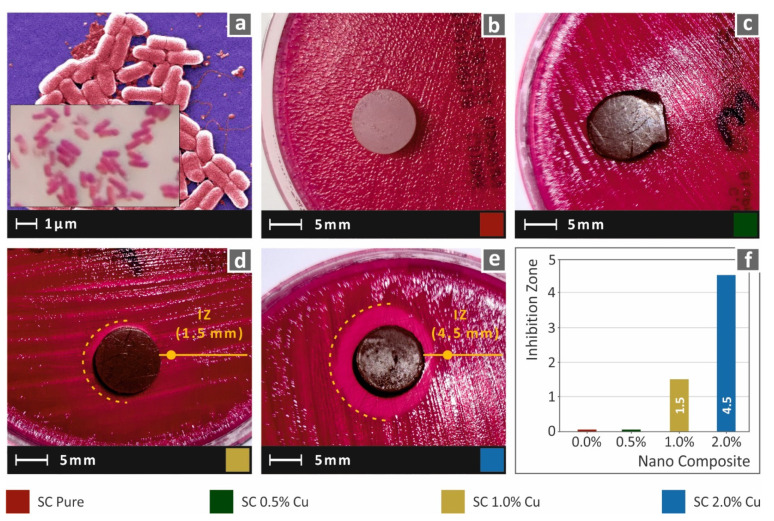
(**a**) typical *E. Coli* morphology, (**b**–**e**) Vertical captures after 24 h cultivation of tested specimen in Petri dish for each corresponding tested material, (**f**) Comparative graph of the measured inhibition zones to filler loading (wt.%).

**Figure 13:**
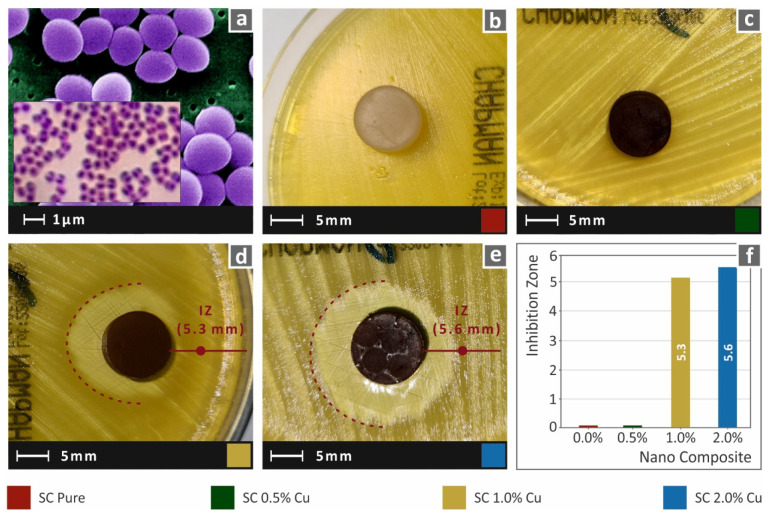
(**a**) typical *S. Aureus* morphology, (**b**–**e**) Vertical captures after 24 h cultivation of tested specimen in Petri dish for each corresponding tested material, (**f**) Comparative graph of the measured inhibition zones to filler loading (wt.%).

In this work, to imitate the biocidal properties of HDPs, copper nanoparticles were used as fillers in commercial SLA resin. While lower filler ratios could not provide results with clearly visible antibacterial performance with the method employed in this work, the SC Cu 2.0 wt.% showed an intense inhibition zone, which was measured approximately 4.5 mm wide. A narrow inhibition zone was observed for the nanocomposite with 1.0 wt.%, while 0.5 wt.% could not provide visible antibacterial activity for gram-negative *E. Coli* bacteria. Images acquired after 24 h of cultivation of *E. coli* for each tested material are shown in Figure 12. Similar behavior was also observed for gram-positive *S. aureus* bacteria, as shown in Figure 13. In this case, the nanocomposites filled with 1.0 wt.% and 2.0 wt.% created a similar inhibition zone of approximately 5.5 mm. Correspondingly, the neat SC and SC Cu 0.5 wt.% nanocomposite did not show antibacterial activity for *S. aureus* bacterium. The filler ratio for introducing antibacterial performance on the specific nanocomposites exhibits a threshold of 1.0 wt.% loading as per the two (2) assessed bacteria. In lower ratios, Cu concentration on the specimens’ surface is not adequate to repel bacterial growth.

## 4. Discussion

In this study, a common resin for the SLA process was used as a matrix material for the development of nanocomposites mimicking HDPs’ biocidal properties. To achieve that, copper, in nanoparticles form, was used as filler in various concentrations. The nanocomposites developed showed at the same time enhanced mechanical response when compared with the matrix material. All mechanical tests conducted in this work, i.e., tensile, flexural, and impact tests, showed a similar trend in the results on the effect of Cu nanoparticles on the matrix material. Cu nanoparticles showed an enhanced mechanical performance up to filling ratios of 1.0 wt.%, while degradation occurred over such filler ratios. The morphological analysis provided significant feedback on the processing quality and confirmed the increase of the ductile behavior of the fabricated nanocomposites, while it revealed valuable information for the fracture mechanism on the tensile specimens. The antibacterial activity of the nanocomposites was adequate at higher filler ratios, while the threshold for mechanical performance enhancement was lower, with the 1.0 wt.% nanocomposite depicting both enhanced mechanical response and antibacterial activity.

The 2.0 wt.% filler ratio implicated plausible agglomeration effects, which may have deteriorated the total performance of the material, as its results were comparable with neat SC. The laser spot size of the utilized 3D printer is 85 microns diameter, and a local agglomeration with a size smaller than 10 microns could diffuse the laser beam. Such an effect could provide low-quality polymerization locally in the interface area of the nanoparticles with the matrix material. This plausible low-quality polymerization enables the possibility of lower fusion in either the intra- or interlayer direction. Even in the SC Cu 1.0 wt.% nanocomposite, minor agglomerations were seen, although the mechanical performance was significantly enhanced. In this case, it could be assumed that the agglomeration size, which is affected by the filler ratio, is important for the polymerization process, the interface quality of the nanoparticles, and the matrix material. Further studies could provide more information about the mechanisms and exact threshold points.

The antibacterial performance results, in combination with the corresponding mechanical performance results, generally indicate a high potential for SLA UV-cured resin Cu nanocomposites. It was found that the 1.0 wt.% loading is sufficient composite material. Such nanocomposites can be easily processed in SLA 3D printers, and the fabrication procedure could be implemented without high-end technology mixing equipment. Similar behavior was also observed for the SC Cu 0.5 wt.%, which further enables a prospect for a more in-depth analysis for the threshold, to fabricate resin Cu nanocomposites with enhanced mechanical performance and antibacterial activity. Such optimized nanocomposites can be used in SLA technology for high-end medical applications.

## 5. Conclusions

In this study, an attempt was made to develop SLA 3D printing materials mimicking the biocidal properties of the HDPs, through an affordable process, using a common low-cost commercial resin suitable for this use. Nanocomposites were developed at various concentrations and their antibacterial performance was investigated using the agar well diffusion method for gram-negative *E. coli* and gram-positive *S. aureus* bacteria. The results show high potential for the introduction of Cu nanoparticles in UV-cured resins in SLA implementations. Cu nanoparticles enhanced the mechanical performance of the nanocomposites, while such an enhancement was conducted with low filler ratios of 0.5 and 1.0 wt.%. Additionally, antibacterial activity was found at higher filler ratios, which further confirmed the great potential of the fabricated materials.

Considering the previously mentioned results, in combination with the processing followed during the current study, it should be mentioned that innovative solutions could be prepared, using commonly used equipment. SLA 3D printing, enabling the manufacturing of high-complexity geometries, along with the enhanced nanocomposite properties, is a strong combination for exploitation in medical implementations. Furthermore, the recent pandemic has led to problems in the existing supply chain systems, which are vulnerable in emergencies and 3D printing can also be exploited in such situations. SLA UV-cured resin Cu nanocomposites were evaluated as key materials, as their fabrication could be implemented in local laboratories, and the 3D printed parts could provide solutions to problems locally. The materials produced in this work could further exploit 3D printing usage during the present pandemic in medical- and engineering-related fields which require materials with antibacterial performance and enhanced mechanical response, such as the materials developed in this work. As future work, the antibacterial performance could be further investigated with more advanced methods and the mechanical and antibacterial performance loading threshold could be further optimized by statistical analysis tools and modeling, which were not among the purposes of the current study.

## Data Availability

The data presented in this study are available upon request from the corresponding author.
